# A general covalent binding model between cytotoxic selenocompounds and albumin revealed by mass spectrometry and X-ray absorption spectroscopy

**DOI:** 10.1038/s41598-020-57983-y

**Published:** 2020-01-27

**Authors:** Wenyi Zheng, Rui He, Roberto Boada, Maria Angels Subirana, Tobias Ginman, Håkan Ottosson, Manuel Valiente, Ying Zhao, Moustapha Hassan

**Affiliations:** 1 Experimental Cancer Medicine, Clinical Research Center, Department of Laboratory Medicine, Karolinska Institute, Huddinge, 141 86 Sweden; 2 Centre GTS, Department of Chemistry, Autonomous University of Barcelona, Barcelona, 08193 Spain; 3 Sprint Bioscience, Huddinge, 141 86 Sweden; 4 Department of Biosciences and Nutrition, Karolinska Institute, Huddinge, 141 86 Sweden; 5 ECM, Clinical Research Center and Center for Allogeneic Stem Cell Transplantation (CAST), Karolinska University Hospital, Huddinge, 141 86 Stockholm Sweden

**Keywords:** Cancer, Chemical biology, Chemistry

## Abstract

Selenocompounds (SeCs) are promising therapeutic agents for a wide range of diseases including cancer. The treatment results are heterogeneous and dependent on both the chemical species and the concentration of SeCs. Moreover, the mechanisms of action are poorly revealed, which most probably is due to the detection methods where the quantification is based on the total selenium as an element. To understand the mechanisms underlying the heterogeneous cytotoxicity of SeCs and to determine their pharmacokinetics, we investigated selenium speciation of six SeCs representing different categories using liquid chromatography-mass spectrometry (LC-MS) and X-ray absorption spectroscopy (XAS) and the cytotoxicity using leukemic cells. SeCs cytotoxicity was correlated with albumin binding degree as revealed by LC-MS and XAS. Further analysis corroborated the covalent binding between selenol intermediates of SeCs and albumin thiols. On basis of the Se-S model, pharmacokinetic properties of four SeCs were for the first time profiled. In summary, we have shown that cytotoxic SeCs could spontaneously transform into selenol intermediates that immediately react with albumin thiols through Se-S bond. The heterogeneous albumin binding degree may predict the variability in cytotoxicity. The present knowledge will also guide further kinetic and mechanistic investigations in both experimental and clinical settings.

## Introduction

Selenocompounds (SeCs) refer to the inorganic and organic compounds containing the selenium (Se) element. Currently, a great number of SeCs are being used in preclinical and clinical investigations. On basis of the inverse relationship between selenium abundance *in vivo* and risk of cancer revealed by observational studies^1^, several clinical trials are ongoing to assess cancer-preventive role of SeCs in human^2–5^. SeCs are also applied to attenuate toxicity from environmental pollutants like arsenic trioxide and improve the health status^6,7^. The rationale behind these applications is largely attributed to the antioxidant functions of selenoproteins, which utilize selenium as a key element in form of selenocysteine. In contrast, SeCs at doses exceeding nutritional level could produce abundant concentrations of reactive oxygen species that induce oxidative damage. The oxitative stress has been utilized in several indications including malignancy, malaria and bacterial infections^8,9^. Interestingly, upregulation of antioxidant status has been identified to underly chemoresistance in cancer, thus the use of SeCs could re-sensitize resistant cancer cells to chemotherapy^10,11^. Noteworthy, the profound effects of SeCs are further encouraging endeavors to improve the efficacy of existing small molecule drugs and antibodies by incorporating selenium element^12,13^.

Despite several investigations, the biological functions of SeCs remain unclearly defined and contradictory results are reported^14,15^. To assess disease preventive potentials of SeCs, two large clinical trials, NPC (Nutritional Prevention of Cancer) and SELECT (Se and vitamin E Cancer prevention Trial), were conducted consecutively. The results were rather inconclusive. One of the plausible reasons is ascribed to the different SeCs used in each trial (selenized yeast containing a mixture of SeCs *vs* selenomethionine)^16,17^. Also, the diverse metabolites from the same SeC might behave uniquely, therefore it is imperative to perform selenium speciation and quantify the species of interest specifically, rather than merely measure the total selenium element, to achieve optimal preventive/therapeutic outcome. Nonetheless, most available methods, including those applied in clinical trials, are based on intensive sample decomposition and subsequent measurement of total selenium element without distinguishing the parent compound from its metabolites^18–20^.

Hyphenated mass spectrometry is the most common tool to perform selenium speciation^19^. It features high sensitivity to detect selenium concentration of PPB (part per billion), low running cost and well simplicity in data interpretation, but the sample preparation prior to data acquisition is usually complex and the information obtained is limited. Moreover, species transformation during sample preparation could not be avoided, making data interpretation being bias-prone. On the contrary, X-ray absorption spectroscopy (XAS) is an advanced technique requiring minimal sample preparation compared to hyphenated techniques. Most interestingly, not only could possible species in complex matrix like cell lysate be identified, also more precise atomic environment of selenium is elucidated^21^. Routine use of XAS is, however, infeasible due to its limited instrumentation and high running cost.

In order to facilitate translational application of SeCs in human, we aimed to perform selenium speciation in human plasma using both liquid chromatography-mass spectrometry (LC-MS) and XAS. Noteworthy, most SeCs currently under investigation are derived from structural modification of precursor compounds and have typical functional groups such as monoselenide, selenocyanate, seleninic acid, diselenide and isoselenazolone. In the present study, we selected six SeCs representing different classes of SeCs to gain an overview.

## Experimental Section

### Reagent

Selenomethionine (SeMet), methylselenocysteine (MeSeCys), methylseleninic acid (MeSeA), N-ethylmaleimide (NEM), tris(2-carboxyethyl) phosphine (TCEP), and human serum albumin (HSA, Cat. No. A1653) were purchased from Sigma-Aldrich (St. Louis, USA). p-XSC (p-xyleneselenocyanate) was obtained from Abcam (Cambridge, UK). Ebselen was purchased from Enzo Lifesciences (Farmingdale, USA). Selenocystine (CysSe_2_) was obtained from SantaCruz (Dallas, USA). Dimethyl acetamide (DMA) and acetonitrile (ACN) were purchased from Merck (Darmstadt, Germany). Dulbecco’s modified eagle’s medium (DMEM), Dulbecco’s phosphate buffer saline (DPBS), penicillin-streptomycin, fetal bovine serum (FBS), and bovine serum albumin (BSA) were obtained from Thermo Fisher (Waltham, USA). C1498 cell line was obtained from ATCC (Manassas, USA) and the cells were luciferase transfected by PerkinElmer (Waltham, USA). Deionized water was produced by Purelab Ultra system from ELGA Veolia with resistance higher than 18.2 MΩ. Blank human plasma was purchased from blood centers as pooled plasma from unidentified healthy individuals and stored at −20 °C until use.

### Synthesis of selenol derivative

Selenol derivative of each SeC (SeC-NEM) was synthesized as depicted in Supplementary Fig. 1. Briefly, SeC solution was mixed with NEM, to which TCEP was added drop-wise under vortexing. The crude derivative was obtained after incubation at room temperature for 30 min and was analyzed using high performance liquid chromatography (HPLC) before purification using preparative liquid chromatography. We observed the emergence of a new peak in parallel to the disappearance of the peak corresponding to the parent compound (data not shown), which suggests complete conversion. The preparative liquid chromatography was conducted on a Gilson HPLC system with a UV detector (detection wavelength of 254 nm). To optimize the separation procedure,  a symmetry or Xterra Prep C18 column was used. The mobile phase consisted of ACN/H_2_O at a flow rate of 35 mL/min. A total of 4–5 fractions (1 ml per fraction) were collected and dried. The derivative was then characterized using ^1^H-NMR (nuclear magnetic resonance, Bruker DRX-400, 400 MHz) and ESI-MS (electrospray ionization-mass spectrometry, Thermo TSQ Quantum Ultra).

### Synthesis of selenol-albumin conjugate

SeC solution (40 µL; 2 mg/mL) was mixed with HSA (50 mg/mL in H_2_O; 4 mL) and kept at room temperature for 30 min. The crude material was separated on a Superdex 200 Increase 10/300 GL column (GE Healthcare). The mobile phase consisted of ACN/H_2_O (5/95; v/v) at a flow rate of 0.75 mL/min. A total of 40 fractions (1 ml per fraction) approximately were collected, pooled and lyophilized (selenol-human serum albumin conjugate; SeC-HSA).

### Analysis of SeC in parent form using deproteinization assay

SeCs were dissolved in either H_2_O (for CysSe_2_, MeSeA, MeSeCys, and SeMet) or DMA (for ebselen and p-XSC). The spiked matrix was obtained by adding 10 µL of SeC solution into 10 µL of blank plasma or 5% BSA solution. To extract the parent SeC from the prepared spiked matrix (20 µL), 50 µL of ACN were added followed by rigorous vortexing. The mixture was centrifuged (30,000 g, 10 min) to obtain the extract. Two µL of the extract were injected into the liquid chromatography-mass spectrometry (LC-MS, Thermo TSQ Quantum Ultra) analyzer. Details about LC conditions (column, mobile phase and flow rate) and MS parameters were listed in Supplementary Table 1.

To clarify the role of albumin thiols, 10 µL of 5% BSA solution were pre-incubated with NEM (10 µL, 0.4 M in DMA) for 10 min before being mixed with SeC solution (10 µL). Again, 50 µL of ACN were used for extraction and 2 µL of the extract were injected into LC-MS for analysis.

### Analysis of SeC using RECID

Blank plasma (10 µL) was spiked with 5 µL of SeC solution. NEM (10 µL, 0.4 M in DMA) and TCEP (5 µL, 0.2 M in H_2_O) were successively added to the spiked plasma. The mixture was vortexed for 30 sec and left for 10 min at room temperature before deproteinization using 50 µL of ACN. Two µL of processed sample were injected for LC-MS analysis (LC-MS parameters listed in Supplementary Table 2).

### Determination of albumin binding degree by ultrafiltration

Two hundred µL of SeC solution were mixed with equal volume of H_2_O (control) or 5% BSA solution (final SeC concentration being 20 µg/mL). The mixture was transferred into an ultrafiltration tube (cut off 10 kDa, Sigma-Aldrich, 13239E) and centrifuged (2000 g and 5 min) to obtain the ultra-filtrate. Twenty µL of the ultra-filtrate were mixed with 50 µL of ACN and analyzed for SeC in parent form. Albumin binding degree was calculated as the percentage of SeC peak area in BSA solution in relative to that in H_2_O.

### X-ray absorption spectroscopy

The mixture of SeC and human plasma (SeC-HP) was prepared. Generally, SeC solution (20 µL; 2 mg/mL) was mixed with 2 mL human plasma, kept at room temperature for 10 min and lyophilized. Selenium K-edge XAS measurements were performed in QEXAFS mode using a Si(311) double-crystal monochromator in CLAESS beamline at ALBA synchrotron light facility (Barcelona, Spain). Self-supported 5 mm pellets of SeC-HSA or SeC-HP (~20 mg) were measured in fluorescence mode using a 5 elements Silicon drift detector. For the reference compounds, the appropriate amount of pure SeC was mixed with cellulose to form pellets with an optimum absorption jump in transmission mode. Transmission was measured with ionization chambers filled with appropriate mixtures of N_2_ and Kr gases. To avoid radiation damage, all measurements were performed at liquid N_2_ temperature using the cryostat available at the beamline^22^. Data analysis was performed using Demeter software package (version 0.9.26)^23^.

### Cytotoxicity of SeC

C1498 cells were cultured in DMEM supplemented with FBS (10%, v/v), penicillin (100 unit/mL) and streptomycin (100 µg/mL). Cells under the exponential growth phase were used for the cytotoxicity study. The stock solutions of SeCs were all of 1 mg/ml, and the solvent was either DMSO (for ebselen and p-XSC) or DPBS (for SeMet, MeSeCys, MeSeA and CysSe_2_, pH adjusted till approximately 7.4 for MeSeA and CysSe_2_). The stock solutions were diluted with cell culture medium till target concentration range. Briefly, 350 µL of cell suspension (0.2 × 10^6^ cells/mL) were mixed with equal volume of SeC solution. The mixture was aliquoted to 96-well plate (100 µL per well) and incubated for 24 hours. Cell viability was determined using CellTiter-Glo kit (Promega, G7571). Cells treated with the appropriate solvent were used as the control. Cell survival rate was calculated as the percentage of absorbance in SeC-treated cells/control group.

### Pharmacokinetic study in mouse

All animal experiments were approved by the Stockholm Southern Ethical Committee and performed in accordance with Swedish Animal Welfare Law (ethical permit no. ID 1031). CysSe_2_ (0.7 mg/mL) or MeSeA (1 mg/mL) was prepared in DPBS and the pH was adjusted to around 7.4. Ebselen or p-XSC was first dissolved in DMSO/Cremophor EL (1:1, v/v) at 5 mg/mL and then diluted with DPBS till 0.5 mg/mL. CysSe_2_ or MeSeA was injected into C57BL/6 albino mouse via the tail vein at a dose of 5 mg/kg; while the dose for ebselen and p-XSC was 2 mg/kg. Approximately 50 µL of blood were collected from the facial vein at different time points into EDTA-coated vials. SeC concentration in plasma was analyzed as described under section “Analysis of SeC using RECID”. The detected concentrations were fitted to a bolus injection in a one-compartment open model and the pharmacokinetic parameters were calculated using WinNonLin software (version 2.0).

## Result

### Albumin binding of selenocompounds

**Figure 1:**
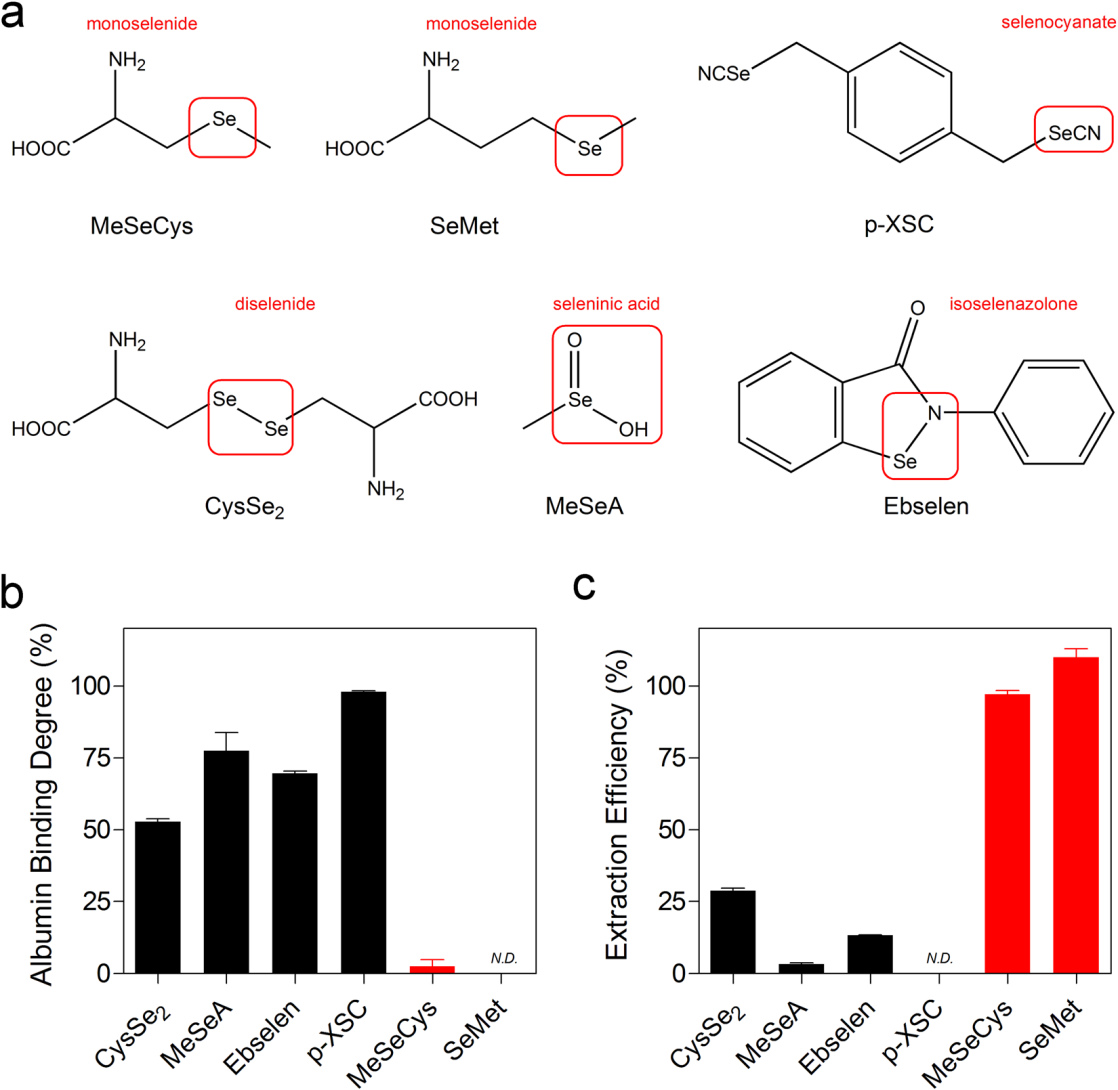
Albumin binding ability of SeC. (**a**) Structure and characteristic functional group of SeC. (**b**) Albumin binding degree of SeC in 5% albumin solution using ultrafiltration method. (**c**) Extraction efficiency of SeC from 5% albumin solution after deproteinization. SeC concentrations were all 20 µg/mL. *N.D*. refers to not detected. Results are shown as the mean ± SD of three technical replicates.

Protein binding is an important issue in the clinical settings since it determines drug availability for distribution to the site of action. To acquire an overview of the plasma protein binding pattern, six SeCs containing different functional groups were selected to represent the broad spectrum of SeCs (Fig. 1a). Initially, SeCs were extracted from plasma through direct deproteinization and the concentrations were measured utilizing LC-MS (Supplementary Table 1). Matrix effect on ionization is commonly seen in the interface of LC and ESI-MS^24^. Under the LC-MS conditions defined in Supplementary Table 1, CysSe_2_ and MeSeA were detected with an eluting time of 2.5 and 4.2 min, respectively (Supplementary Fig. 2). However, their peaks were within the range of ionization suppression by plasma extracts (2.0–2.9 min for CysSe_2_ and 3.0–7.6 min for MeSeA), indicating that direct quantification of CysSe_2_ and MeSeA in plasma was unreliable. Since albumin is the major plasma protein, we used 5% BSA solution as the matrix instead of plasma to elucidate the binding pattern. No matrix effect on ionization of CysSe_2_ and MeSeA was observed when BSA solution was used (Supplementary Fig. 2), thus accurate quantification could be achieved.

Low degree of albumin binding (<3%) was observed for MeSeCys and SeMet using ultrafiltration assay, while CysSe_2_, MeSeA, ebselen and p-XSC demonstrated high degree of albumin binding ranging 52–100% (Fig. 1b). Deproteinization is the most common method to extract drugs in free and non-covalently bound forms from plasma prior to analysis. Herein, after deproteinization of BSA solution, we were able to extract MeSeCys and SeMet with an overall efficiency over 95%. However, the extraction efficiency for the other four SeCs was as low as 0–28% (Fig. 1c). Briefly, SeC was poorly available in free form or non-covalently bound form, covalent interaction was thus suspected to contribute to the strong binding ability.

### Investigation of the binding using X-ray absorption spectroscopy

**Figure 2:**
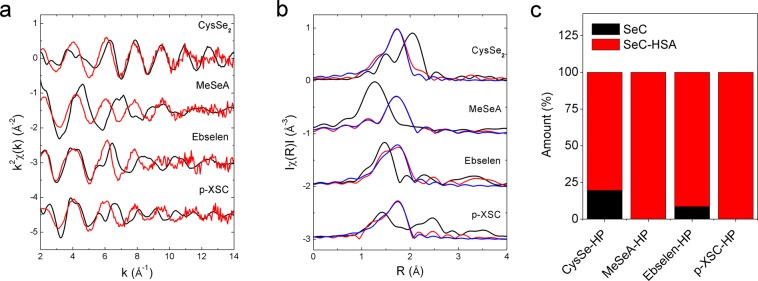
X-ray absorption spectroscopy measurement. (**a**) Extended X-ray absorption fine structure (EXAFS) spectra of SeC (black), SeC-HSA (red). (**b**) Fourier transform of EXAFS of SeC (black), SeC-HSA (red). Result from EXAFS fitting of SeC-HSA assuming Se-S bond was shown in blue line. Distances were not phase shift corrected. (**c**) Amount of SeC and SeC-HSA in SeC-HP estimated from the linear combination fitting analysis of the XANES spectra. The total concentration of SeC in SeC-HP was 20 µg/mL.

In order to confirm the albumin binding, SeC was firstly allowed to react with HSA. The crude material was then separated by size exclusion chromatography and the eluent corresponding to free HSA was collected and lyophilized (SeC-HSA). SeC-HSA was subsequently subjected to XAS measurement and compared to the SeC itself. The signals corresponding to selenium in the commercial HSA were negligible compared to that in SeC-HSA (<0.1%) and thus would not interfere with interpretation of the readout in our study. Both regions of the XAS spectra, X-ray absorption near edge spectrum (XANES) and extended X-ray absorption fine structure (EXAFS), for SeC-HSA were markedly different from that of the corresponding SeC (Supplementary Fig. 3; Fig. 2a), indicating a change in the coordination environment of Se. Noteworthy, the EXAFS signal was similar for all the four SeC-HSA conjugates (Fig. 2a). The pseudo-radial distribution function obtained after Fourier transform of the EXAFS clearly showed a characteristic feature which was common to all SeC-HSA conjugates (Fig. 2b). Further modeling of the pseudo-radial distribution function of SeC-HSA supported the hypothesis that Se coordinates with S (sulfur; Table 1). In addition, since the XAS spectra of mixtures can be expressed as an additive combination of its constituents, a linear combination fitting analysis using the reference spectra of SeC-HSA and SeC was carried out to assess the amount of each form in the mixture of SeC and human plasma (SeC-HP). Results showed that SeC-HSA was a major constituent in SeC-HP (Fig. 2c, Supplementary Table 3). Conclusively, XAS analysis confirmed the formation of Se-S bond and demonstrated notable albumin binding of SeCs in context of human plasma.

**Table 1:** Coordination of selenium atom in selenol-human serum albumin conjugate.

SeC-HSA	Atom	Number	E_0_ (eV)	R (Å)	σ^2^ (Å^2^)	R-factor
CysSe-HSA	C	1	6.7	2.016	0.003	0.047
	S	1		2.191	0.001	
MeSeA-HSA	O	1	5.8	1.745	0.030	0.004
	S	1		2.178	0.002	
Ebselen-HSA	C	1	7.0	1.957	0.002	0.024
	S	1		2.205	0.002	
p-XSC-HSA	C	1	6.9	1.972	0.009	0.036
	S	1		2.190	0.003	

The analysis was obtained from the modelling of the EXAFS signal. The k-range used was 2.6–13.3 Å^−1^ except for MeSeA-HSA for which the upper bound was 10.1 Å^−1^. The amplitude reduction factor (S_0_^2^) was fixed to 0.85 after fitting a reference of sodium selenite.

### Role of selenol and thiol groups in albumin binding

**Figure 3:**
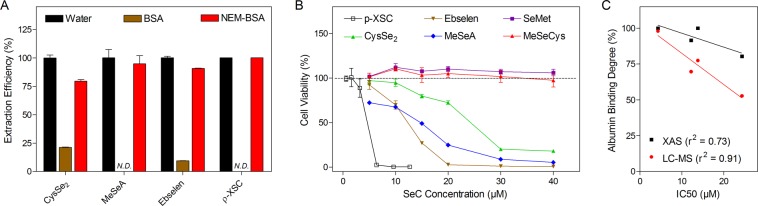
Role of selenol and thiol in albumin binding. (**A**) Extraction efficiency of SeC in H_2_O, 5% BSA, and NEM pretreated-BSA solution after deproteinization. SeC concentrations were 20 µg/mL. *N.D*. refers to not detected. Results are shown as the mean ± SD of three technical replicates. (**B**) Cell viability of C1498 cells after SeC treatment for 24 hr. Results are shown as the mean ± SD of six biological replicates. (**C**) Relationship between the half-inhibitory concentration (IC50) and albumin binding degree of the four cytotoxic SeCs. IC50 was calculated from the experiments related to panel *b*. Albumin binding degree was extracted from the LC-MS (Fig. 1b) or XAS (Fig. 2c) analysis. The goodness of linear regression fitting is shown as r^2^.

The binding site of SeCs on HSA most likely is located at the Cys34 residue which is easily accessible and highly reactive according to what was reported previously^25^. To further understand how the Se-S bond was formed, we used excess thiol-reactive agent NEM to eliminate the effect of the thiol groups. Compared to pristine albumin, higher extraction efficiency of the parent compound (CysSe_2_: 79.6% *vs* 21.4%; MeSeA: 94.8% *vs* 0%; ebselen: 90.7 *vs* 9.3%; p-XSC: 100% *vs* 0%) was obtained after blocking albumin thiols, which suggests the absence of binding between SeC and NEM-treated albumin (Fig. 3A).

On the other hand, we assessed the cytotoxicity of SeCs which had been positively correlated with generation of selenol^26^. As shown in Fig. 3B, only the compounds with prominent albumin binding (i.e. CysSe_2_, MeSeA, ebselen and p-XSC) showed cytotoxicity on the C1498 leukemic cells; whereas no cytotoxic effect, within the concentration range tested, was observed for the compounds that did not exhibit albumin binding (i.e. MeSeCys and SeMet). Similar cytotoxic pattern was observed in the murine breast cancer cell line 4T1 and human leukemia cell line HL60 (data not shown). Moreover, a negative correlation was found between the IC50 and albumin binding degree acquired from both XAS and LC-MS measurements (Fig. 3C).

**Figure 4:**
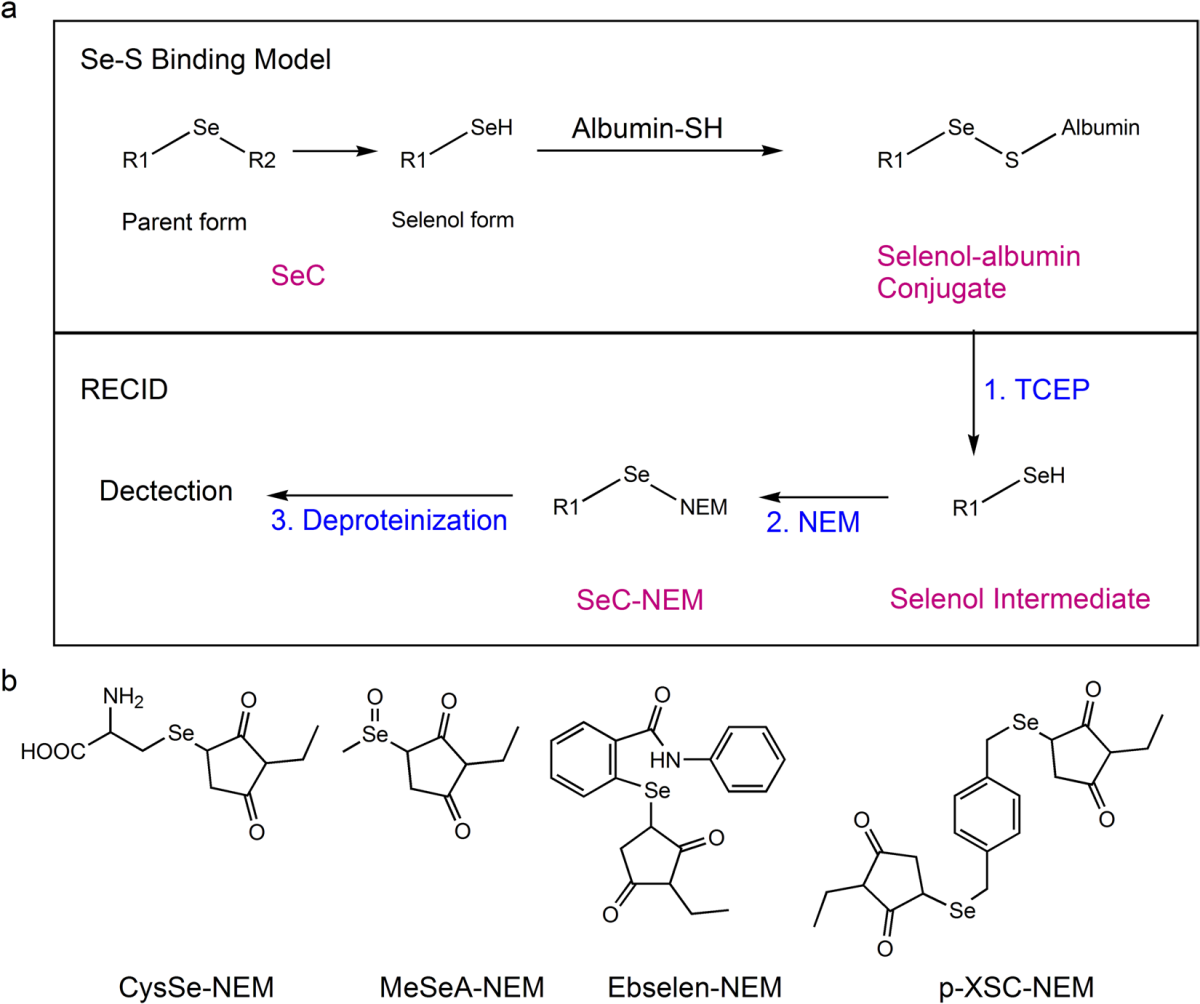
Formation of selenol by cytotoxic SeC. (**a**) Scheme of Se-S binding model and reductive cleavage and instant derivatization method (RECID). (**b**) Chemical structure of selenol derivative (SeC-NEM) of each SeC.

In our previous investigation on p-XSC^27^, we had established a REductive Cleavage and Instant Derivatization method (RECID) to quantify both free and albumin-bound forms. RECID contains three consecutive steps: (1) generation of selenol intermediate by TCEP; (2) derivatization of the selenol intermediate by NEM; (3) final extraction of the derivative by deproteinization (Fig. 4a). We leveraged RECID to track how selenol was formed. The speculated selenol derivative of each SeC (SeC-NEM) was synthesized as described in Supplementary Fig. 1. The characterization using ESI-MS showed the molecular ion (m/z = 294.9 for CysSe-NEM; 291.9 for MeSeA-NEM; 402.9 for ebselen-NEM) and isotopic pattern for each derivative (Supplementary Fig. 4). In the ^1^H-NMR spectra, there was a common peak at around 1 ppm that corresponds to methyl proton (Supplementary Fig. 5). This peak most certainly originated from NEM since none of the parent SeCs has a methyl group. On the basis of the results above, the structures of SeC-NEM (Fig. 4b) and stoichiometry of SeCs versus albumin thiol (Supplementary Table 4) could be concluded. Collectively, cytotoxic SeC could spontaneously transform into selenol and immediately reacts with albumin thiol through Se-S bond.

### Utilization of the binding model for profiling pharmaceutics

On top of the binding model established above, we asked whether RECID was generally amenable to quantify cytotoxic SeC in plasma. To achieve high yield, the reaction time, the amount of TCEP and NEM as well as the sequence in addition of TCEP/NEM were optimized as previously reported (Supplementary Fig. 6)^27^. Noteworthy, the reactions were all found to reach their maximum within less than 1 min, indicating efficient conversion. Using the optimized RECID and LC-MS conditions (Supplementary Table 2), our method achieved high specificity in plasma (Supplementary Fig. 7). The results of methodological validation further ensured accurate quantification using the RECID method (Supplementary Tables 5 and 6). The spiked sample and processed sample were stable at usual storage conditions, allowing for routine quantification procedures (Supplementary Table 7). Furthermore, switching human plasma into mouse plasma had few effects on the quantification results (Supplementary Fig. 8), which indicates the robustness of RECID in different matrices.

**Figure 5:**
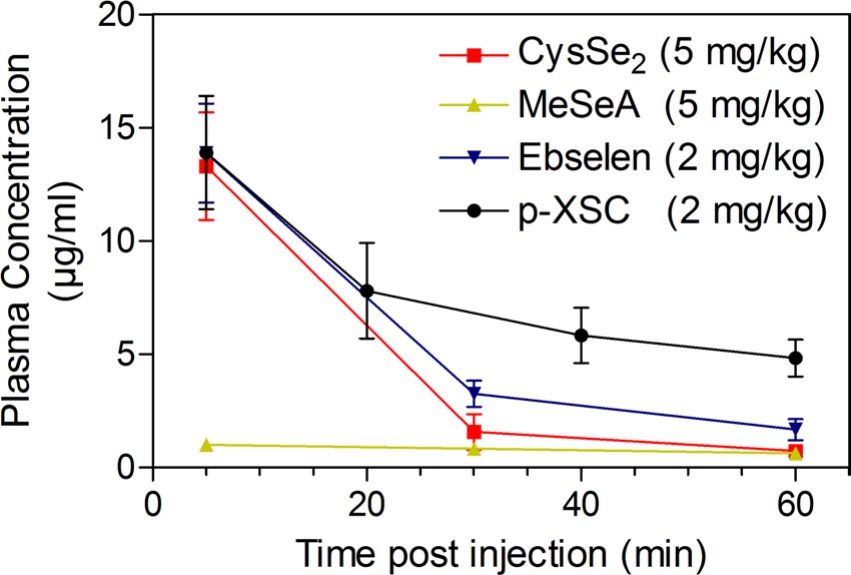
Concentration-time curve of SeC in mouse after intravenous injection. Blood samples were taken at different time points post SeC administration and analyzed using RECID. Results are shown as the mean ± SD of three mice for each compound.

After intravenous administration of CysSe_2_, ebselen or p-XSC, similar concentration-time curves were observed (Fig. 5). Since CysSe_2_ was given at a 2.5-fold higher dose compared to ebselen and p-XSC, its disappearance from plasma was deemed much faster. Moreover, although MeSeA was injected at the same dose as CysSe_2_, its level at the first time point (5 min post injection) was much lower followed by a flat curve. Pharmacokinetic modeling of the concentration-time curve was performed except for MeSeA the curve of which could not be modeled. As can be seen in Table 2, p-XSC showed the lowest clearance rate (5.8 mL/h), the longest half-life (30.6 min) and mean retention time (44.4 min), and conferred the highest exposure (10.8 µg/mL·h). The total exposure of CysSe_2_ was around 72% compared to that of ebselen; if adjusted for dose, its exposure was less than one third of that of ebselen.

**Table 2:** Pharmacokinetic parameter of SeC in mouse.

Parameter	CysSe_2_ (5 mg/kg)	MeSeA (5 mg/kg)	Ebselen (2 mg/kg)	p-XSC (2 mg/kg)
AUC (µg/mL·h)	4.1 ± 1.1	*N.A*.	5.7 ± 0.7	10.8 ± 2.4
C_max_ (µg/mL)	20.4 ± 2.7	*N.A*.	18.1 ± 3. 7	14.8 ± 2.7
Cl (mL/h)	38.5 ± 11.7	*N.A*.	10.6 ± 1.3	5.8 ± 1.3
K10-HL (min)	8.4 ± 1.2	*N.A*.	13.2 ± 2.4	30.6 ± 7.2
MRT (min)	12 ± 2.4	*N.A*.	19.2 ± 3.6	44.4 ± 10.2
V_ss_ (mL)	7.5 ± 1.0	*N.A*.	3.4 ± 0.6	4.1 ± 0.8

SeC concentration-time curve was fitted into one-compartment open model to calculate pharmacokinetic parameters. Results are shown as mean ± SD of three mice per compound. *N.A*. refers to not available. AUC: area under the curve; C_max_: peak concentration; Cl: rate of clearance; K10-HL: elimination half-life; MRT: mean retention time; V_ss_: apparent volume of distribution at steady state. *N.A*. refers to not available.

## Discussion

During the last decade, SeCs have gained significant attention both as preventive and treatment agents for a plethora of diseases including cancer. Today, SeCs are present in several human daily supplements as well as in several clinical trials^1^. Accumulating lines of evidences have highlighted critical impact of the form and concentration of SeCs on their biological functions. Thus, speciation and quantification of SeCs in biological matrices and further elucidation of both their pharmacokinetic properties *in vivo* and their mechanism of action have become of high priority. However, the available knowledges are predominately based on measurement of total selenium element without distinguishing the parent compounds from their metabolites^28–30^. The present study combined hyphenated mass spectrometry and X-ray absorption spectroscopy for selenium speciation and uncovered a general covalent binding model between cytotoxic SeCs and albumin, irrespective of physiochemical attributes in terms of structure and hydrophilicity (Fig. 4). Sugio *et al*. have reported that Cys34 is the only free thiol residue on HSA, therefore we assume that the binding site for SeC is likely to be Cys34 residue^25^; however, other residues cannot be ruled out. The established model was further translated into pharmacokinetic profiling of four diverse SeCs using a derivatization method measuring both the free and albumin-bound fractions (Fig. 5). In our article published recently, albumin was found to be crucial in the determination of SeCs cytotoxicity^31^. Noteworthy, during several disease conditions, albumin concentrations are highly altered in many patients. Such albumin status will certainly have a high impact on treatment efficacy, toxicity and adverse effects of SeCs, which in turn highlights the importance of quantification of albumin-bound fractions.

Our results marked a clear correlation between SeC cytotoxicity and albumin binding degree most probably because both are dependent on the intrinsic ability of SeC to transform into selenol (Fig. 4)^26^. Despite that SeMet and MeSeCys showed neither cytotoxicity nor albumin-binding ability in this study, addition of an appropriate lyase was reported to switch on their cytotoxic facets^32,33^. Hence albumin-bound selenol species should be investigated in pursuit of their active metabolites. More broadly, emerging cytotoxic or chemotherapeutic SeCs are presumably predisposed to strong albumin binding ability and RECID could be used for their quantification. Lastly, for hydrophilic selenocompounds like CysSe_2_ and MeSeA, although changing the chromatography conditions could relieve the adverse matrix effect on compound ionization (Supplementary Fig. 2) through better separation, matrix effect on extraction is still not addressed due to the presence of strong albumin binding. However, the present analytical method (RECID) has resolved the poor separation as well as the albumin binding issue of CysSe_2_ and MeSeA.

In dynamic contexts like cells and blood circulation, downstream selenol metabolites might be present including methyl selenol and hydrogen selenide^34^. The high reactivity of selenol *per se* leads to low abundance at steady state and challenges direct measurement^26^. As RECID relies on generation and instant stabilization of selenol intermediates, it might be used for quantification of other selenol metabolites beyond the selenol form of parent compound. Accordingly, depiction of a clearer metabolic pathway could be envisioned. Noteworthy, as albumin is present in various biological matrices (e.g. cell culture medium, tissue homogenate, cell lysate), we believe that albumin binding prevails and ubiquitously interferes with SeC quantification. Accordingly, application of RECID could be broadened from plasma to many other biological contexts. But it is cautionary to consider the maximal amount of TCEP-susceptible bonds (e.g. disulfide) and NEM-reactive groups, and accordingly optimize the TCEP/NEM amount^35^.

Although the four cytotoxic SeCs (i.e. CysSe_2_, MeSeA, ebselen and p-XSC) have received significant attentions in disease management^36–38^, their behaviors *in vivo* remain elusive. Due to the shared feature in albumin binding, their pharmacokinetic properties were all elucidated for the first time using RECID (Fig. 5, Table 2). Ebselen and p-XSC are both highly lipophilic and should theoretically have large distribution volume *in vivo*. Surprisingly, their distribution volume was determined to be 3.4 and 4.1 mL, respectively, being close to blood volume (around 2 mL in adult mouse), supporting significant specie transformation. Secondly, since the four SeCs all have strong binding affinity to albumin, their pharmacokinetic properties were assumed to resemble mouse albumin to some extent. Whereas rather unique pharmacokinetic properties were observed, particularly in case of MeSeA; and they had much shorter half-life compared to I^131^-labelled mouse albumin (<30 min *vs* 21 hr)^39^. Likely, SeC-albumin conjugate was more quickly eliminated by albumin scavenger, but this suspicion requires further investigation. Clearly, more studies are warranted to delineate how albumin binding impacts ADME (absorption, distribution, metabolism and excretion) as well as pharmacological activity of SeC.

In conclusion, we have integrated two independent and complementary strategies, hyphenated mass spectrometry and X-ray absorption spectroscopy, in selenium speciation, the results of which not only revealed a general binding model between cytotoxic SeCs and albumin, also laid the foundation for the pioneering pharmacokinetic investigations of four diverse SeCs.

## Supplementary information

Supplementary information.

## Data Availability

All data generated during this study are included in this published article (and its Supplementary Material file) and available from the corresponding author on reasonable request.
