# Supplementary material for: A general covalent binding model between cytotoxic selenocompounds and albumin revealed by mass spectrometry and X-ray absorption spectroscopy
Source: Sci Rep. 2020 Jan 27;10:1274. doi: 10.1038/s41598-020-57983-y (PMC6985102; doi:10.1038/s41598-020-57983-y)

# Supplementary Material

# A general covalent binding model between cytotoxic selenocompounds and albumin revealed by mass spectrometry and X-ray absorption spectroscopy

Wenyi Zheng[1,5], Rui He[1,5], Roberto Boada[2], Maria Angels Subirana[2], Tobias Ginman[3], Håkan Ottosson[4], Manuel Valiente[2], Ying Zhao[1,5,*], Moustapha Hassan[1, 5,*]

1. Experimental Cancer Medicine, Clinical Research Center, Department of Laboratory Medicine, Karolinska Institute, Huddinge 141 86, Sweden

2. Centre GTS, Department of Chemistry, Autonomous University of Barcelona, Barcelona 08193, Spain

3. Sprint Bioscience, Huddinge 141 86, Sweden

4. Department of Biosciences and Nutrition, Karolinska Institute, Huddinge 141 86, Sweden

5. ECM, Clinical Research Center and Centre for Allogeneic Stem Cell Transplantation (CAST), Karolinska University Hospital, Huddinge, 141 86, Stockholm, Sweden

*. Corresponding Author:

Moustapha Hassan, E-mail address: Moustapha.Hassan@ki.se

Ying Zhao, E-mail address: Ying.Zhao.1@ki.se

ECM, Clinical Research Center,

Karolinska University Hospital, Huddinge

141 86 Stockholm, Sweden

**Supplementary Table 1. LC-MS parameters to measure parent selenocompound.**

| | Item | $CysSe_2$ | MeSeA | Ebselen | p-XSC | MeSeCys | SeMet |
|---|---|---|---|---|---|---|---|
| LC | Column | YMC AQ12S05-1546WT | | | | | |
| | Mobile Phase | ACN/$H_2O$ with 0.1% Formic Acid | | | | | |
| | Flow Rate (ml/min) | 0.6 | 0.4 | 0.4 | 0.2 | 0.4 | 0.4 |
| | ACN Volume (%) | 70% | 70% | 70% | 70% | 30% | 20% |
| | Running Time (min) | 5 | 6 | 8 | 15 | 6 | 7 |
| MS | Spray Voltage (V) | 4500 | 5000 | 5000 | 5000 | 5000 | 5000 |
| | Sheath Gas (Arb) | 50 | 10 | 40 | 50 | 30 | 60 |
| | Auxiliary Gas (Arb) | 5 | 5 | 5 | 5 | 15 | 5 |
| | Ion Sweep Gas (Arb) | 1 | 2 | 2 | 2 | 2 | 2 |
| | Skimmer Offset (V) | -17 | -18 | -2 | -15 | -24 | -16 |
| | Capillary Temperature (°C) | 400 | 297 | 400 | 375 | 312 | 400 |
| | Tube Lens Offset (V) | 100 | 131 | 106 | 100 | 128 | 86 |
| | Collision Energy | 1.2 | N.A. | N.A. | N.A. | N.A. | N.A. |
| | Collision Pressure (mTorr) | -13 | N.A. | N.A. | N.A. | N.A. | N.A. |
| | Ion of Monitor (m/z) | 336.8→247.8 | 128.9 | 275.9 | 209.9 | 166.9 | 197.9 |

Arb: Arbitrary units. N.A.: Not applicable.

**Supplementary Table 2. LC-MS parameters to measure selenol derivative.**

| | Item | CysSe-NEM | MeSeA-NEM | Ebselen-NEM | p-XSC-NEM |
|---|---|---|---|---|---|
| LC | Column | YMC AQ12S05-1546WT | | | |
| | Mobile Phase | ACN/$H_2O$ with 0.1% Formic Acid | | | |
| | Flow Rate (ml/min) | 0.4 | 0.2 | 0.4 | 0.2 |
| | ACN Volume (%) | 20 | 70 | 70 | 70 |
| | Running Time (min) | 11 | 15 | 10 | 20 |
| MS | Spray Voltage (V) | 4500 | 5000 | 4000 | 5000 |
| | Sheath Gas (Arb*) | 50 | 40 | 40 | 40 |
| | Auxiliary Gas (Arb) | 5 | 35 | 5 | 50 |
| | Ion Sweep Gas (Arb) | 1 | 1 | 2 | 1.5 |
| | Skimmer Offset (V) | -17 | -6 | -15 | -15 |
| | Capillary Temperature (°C) | 400 | 400 | 400 | 277 |
| | Tube Lens Offset (V) | 100 | 80 | 75 | 186 |
| | Ion of Monitor (m/z) | 294.9 | 221.9 | 402.9 | 309.9 |

**Supplementary Table 3. Linear combination fitting results of X-ray absorption near edge spectra.** The fitting was performed over the spectra for selenocompound-human plasma mixture between -20 and 50 eV keeping $E_0$ fixed and equal to 0 eV.

| SeC | Total SeC (µg/ml) | SeC (%) | SeC-HSA (%) | R-factor | Chi-square | Reduced Chi-squre |
|---|---|---|---|---|---|---|
| $CysSe_2$ | 20 | 19.6 | 80.4 | 0.002 | 0.050 | 0.0004 |
| MeSeA | 20 | 0 | 100.0 | 0.029 | 0.093 | 0.0068 |
| Ebselen | 20 | 8.5 | 91.5 | 0.004 | 0.103 | 0.0009 |
| p-XSC | 20 | 0 | 100.0 | 0.005 | 0.164 | 0.0012 |

**Supplementary Table 4. Stoichiometry of selenocompound versus albumin thiol.** The selenol intermediate of p-XSC has two selenol groups, but it was unclear whether both could bind to albumin thiol at the same time.

| SeC | Stoichiometry (SeC: Selenol intermediate: Albumin thiol) |
|---|---|
| $CysSe_2$ | 1:2:2 |
| MeSeA | 1:1:1 |
| Ebselen | 1:1:1 |
| p-XSC | 1:1:1 or 1:1:2 |

**Supplementary Table 5. Summary of linearity and limit of detection.** SeC-spiked plasma was analyzed using RECID. Linearity was evaluated by considering the detector response (peak area) to different amounts of SeC by means of linear regression. Results were generated from three technical replicates. Limit of detection (LOD) was defined as signal/noise ratio equal to 3.

| SeC | Linear range (μg/mL) | Slope (Mean ± SD) | Y-Intercept (Mean ± SD) | Correlation coefficient | LOD (μg/mL) |
|---|---|---|---|---|---|
| $CysSe_2$ | 2-40 | 67940 ± 928.7 | 27250 ± 19830 | 0.9990 | 0.5 |
| MeSeA | 1-40 | 96420 ± 803.1 | 30830 ± 16560 | 0.9997 | 0.2 |
| Ebselen | 1-40 | 131100 ± 1941 | -34530 ± 40020 | 0.9980 | 0.2 |

**Supplementary Table 6. Validation of RECID.** SeC-spiked plasma was processed using RECID. Precision was shown as the relative standard deviation of three individual measures of the analyte in multiple aliquots of the same stock solution. Accuracy was the percentage of concentration calculated with calibration curve to the nominal concentration. Recovery was the percentage of peak area of the analyte in plasma to that in solution. Results about accuracy and recovery are shown as the mean ± SD of three technical replicates.

| SeC | | Precision (%) | | Accuracy (%) | Recovery (%) |
|---|---|---|---|---|---|
| Type | Level (μg/mL) | Intra-day | Inter-day | | |
| $CysSe_2$ | 2 | 1.7 | 4.4 | 90.3 ± 1.8 | 88.7 ± 3.9 |
| | 10 | 7.7 | 7.3 | 108.9 ± 8.8 | 78.6 ± 4.0 |
| | 40 | 5.5 | 3.7 | 101.5 ± 5.7 | 79.0 ± 3.8 |
| MeSeA | 1 | 5.6 | 0.9 | 115.3 ± 7.9 | 100.5 ± 0.7 |
| | 10 | 5.5 | 6.7 | 97.3 ± 5.7 | 86.5 ± 1.5 |
| | 40 | 2.6 | 1.4 | 103.0 ± 2.6 | 89.9 ± 0.3 |
| Ebselen | 1 | 3.7 | 2.2 | 107.0 ± 2.9 | 79.9 ± 6.9 |
| | 10 | 0.5 | 0.8 | 107.8 ± 2.5 | 75.3 ± 2.7 |
| | 40 | 3.4 | 3.8 | 103.2 ± 3.5 | 71.5 ± 2.0 |

**Supplementary Table 7. Stability of selenocompound in plasma and selenol derivative in processed sample.** SeC-spiked plasma was treated as indicated and then analyzed using RECID. SeC-NEM in processed sample was treated in the same way and then subjected to LC-MS analysis. Stability was shown as the percentage of the peak area after indicated treatment to that from base line value. SeC concentrations were 20 µg/mL. Results are shown as the mean ± SD of three technical replicates.

| SeC | Sample Type | Treatment | | | |
|---|---|---|---|---|---|
| | | RT/24 hr | 4 °C/24 hr | -20 °C/24 hr | Freeze-Thaw/5 Cycles |
| $CysSe_2$ | Spiked Plasma | 98.6 ± 7.1 | 100.9 ± 7.9 | 99.6 ± 6.4 | 102.2 ± 0.6 |
| | Processed | 104.3 ± 0.4 | 99.1 ± 1.8 | 101.5 ± 4.6 | 97.1 ± 1.9 |
| MeSeA | Spiked Plasma | 110.9 ± 2.0 | 108.9 ± 4.8 | 116.0 ± 3.2 | 117.3 ± 0.5 |
| | Processed | 114.3 ± 0.3 | 106.7 ± 0.5 | 103.2 ± 1.0 | 104.3 ± 0.4 |
| Ebselen | Spiked Plasma | 110.3 ± 1.8 | 113.1 ± 0.5 | 108.2 ± 3.2 | 97.2 ± 2.7 |
| | Processed | 106.8 ± 3.0 | 106.7 ± 0.3 | 108.7 ± 0.7 | 104.5 ± 0.6 |

**Supplementary Figure 1. Synthesis of selenol derivative.** (a) Scheme for synthesis of selenol derivative (SeC-NEM). (b) Formulation for SeC-NEM synthesis.

a

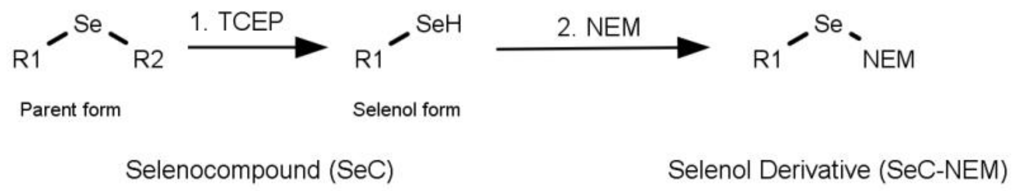


b

| Selenocompound | | | | NEM (20 mg/ml in ACN) | TCEP (50 mg/ml in $H_2O$) |
|---|---|---|---|---|---|
| Type | Solvent | Concentration | Amount | | |
| $CysSe_2$ | $H_2O$; Neutralized | 10 mg/ml | 2 ml | 1 ml | 0.46 ml |
| MeSeA | $H_2O$; Neutralized | 11 mg/ml | 2 ml | 1.5 ml | 1.1 ml |
| Ebselen | ACN | 14 mg/ml | 1 ml | 0.7 ml | 0.34 ml |

**Supplementary Figure 2. Matrix effect on the ionization of selenocystine and methylseleninic acid.** In the upper lane were the chromatograms of $CysSe_2$ and MeSeA injected through LC auto-sampler. Alternatively, to assess matrix effect on analyte ionization, $CysSe_2$ and MeSeA were continuously infused through MS syringe. In the same time, 2 µL of deproteinized extracts from plasma (middle lane) or 5% BSA (lower lane) were injected through LC auto-sampler. The chromatograms were acquired.

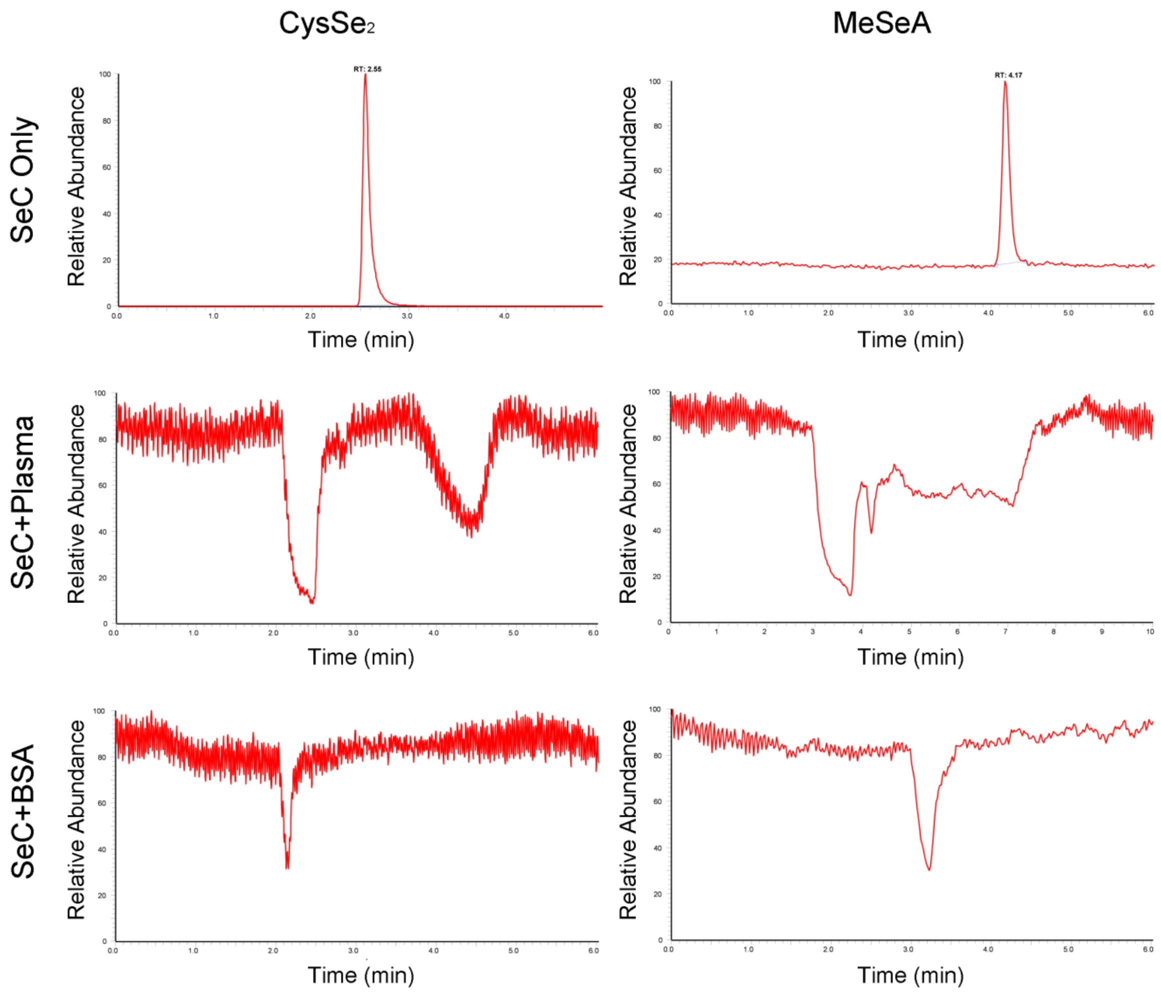

**Supplementary Figure 3. X-ray absorption near edge spectra.** Black, red and green lines indicate SeC, SeC-HSA and SeC-HP, respectively.

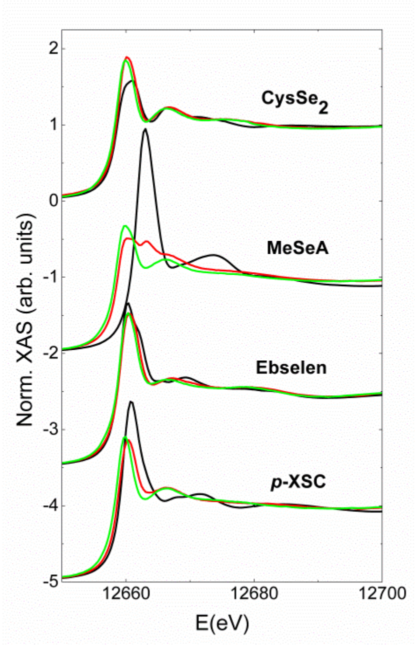

**Supplementary Figure 4. Electrospray ionization-mass spectrometry spectrum of selenol derivative.** SeC-NEM was dissolved in ACN at appropriate concentration and directly infused through MS syringe. The spectrum was acquired using full-scan Q1 mode (Peak Width of 0.7 ).

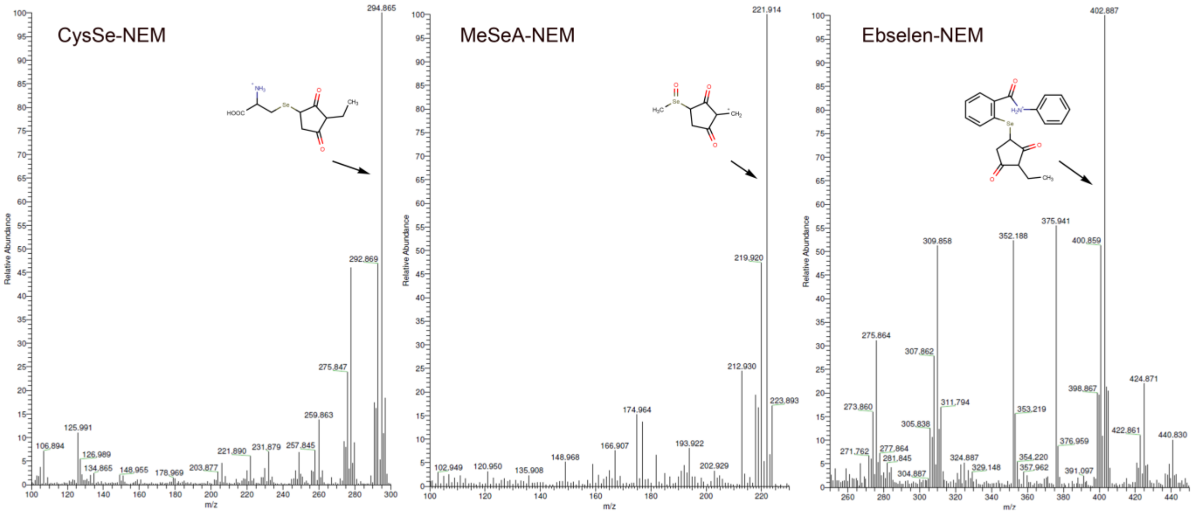

**Supplementary Figure 5. $^{1}$H-NMR spectrum of selenol derivative.** CysSe-NEM and MeSeA-NEM were dissolved in $D_2O$, while Ebselen-NEM was dissolved in $CDCl_3$ for $^1$H-NMR analysis (25 °C, 400 MHz, Bruker DRX-400, Germany).

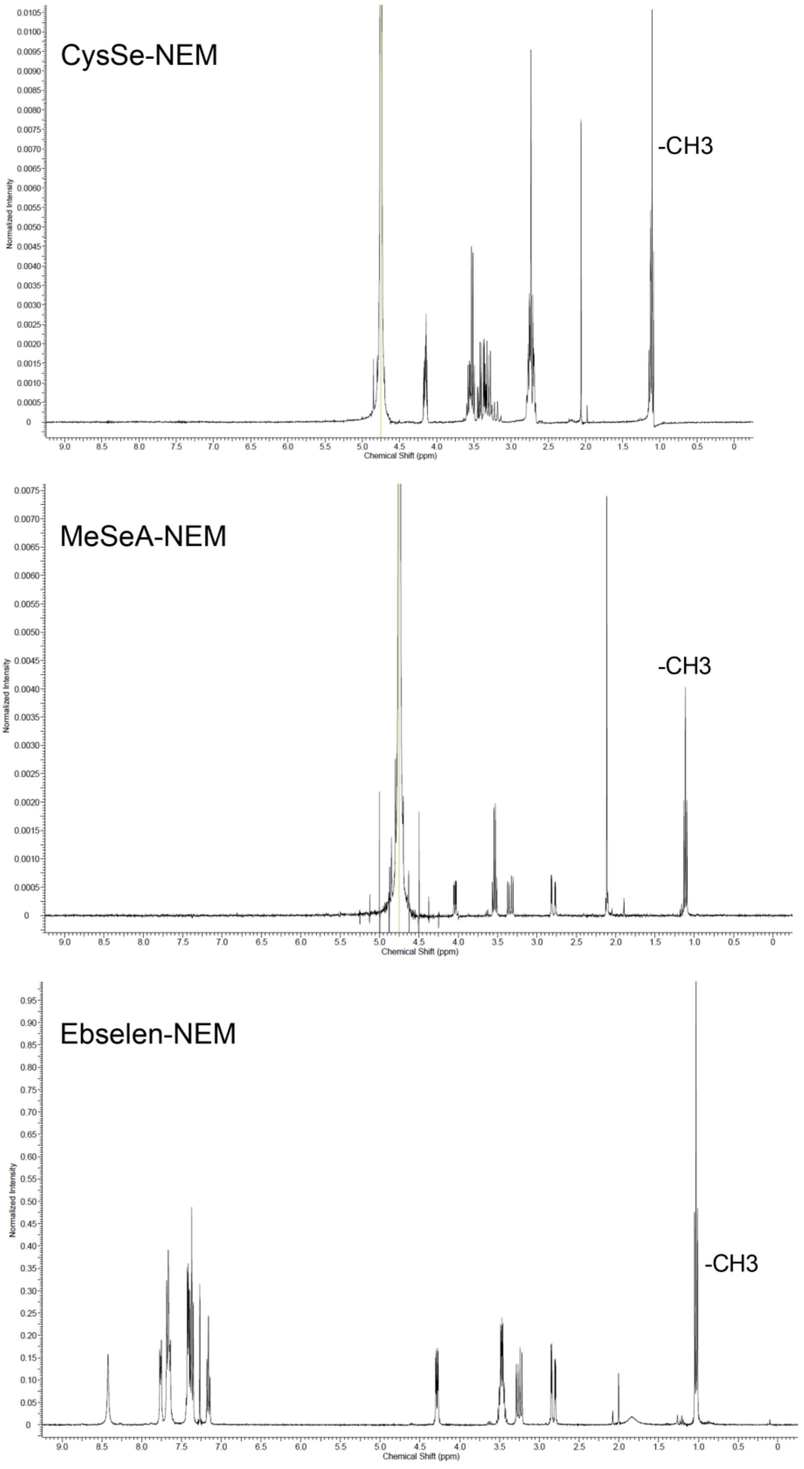

**Supplementary Figure 6. Optimization of RECID.** (a-c) Yield of the derivative after different reaction times. TCEP and NEM amount were 1 μmol and 4 μmol, respectively. (d-f) Yield of the derivative at different TCEP concentrations. NEM concentration was 4 μmol and reaction time was 10 min. (g-i) Yield of the derivative at different NEM concentrations. TCEP concentration was 1 μmol, and the reaction time was 10 min. (j-l) Yield of the derivative upon changing the sequence in adding TCEP (1 μmol) and NEM (4 μmol): TCEP before NEM (1) or NEM before TCEP (2). The reaction time was 10 min. In experiments related to panel a-i, NEM was added before TCEP. In all experiments, derivative yield was shown as the percentage of peak area at indicated setting to that at optimal condition. Results are shown as the mean ± SD of three technical replicates.

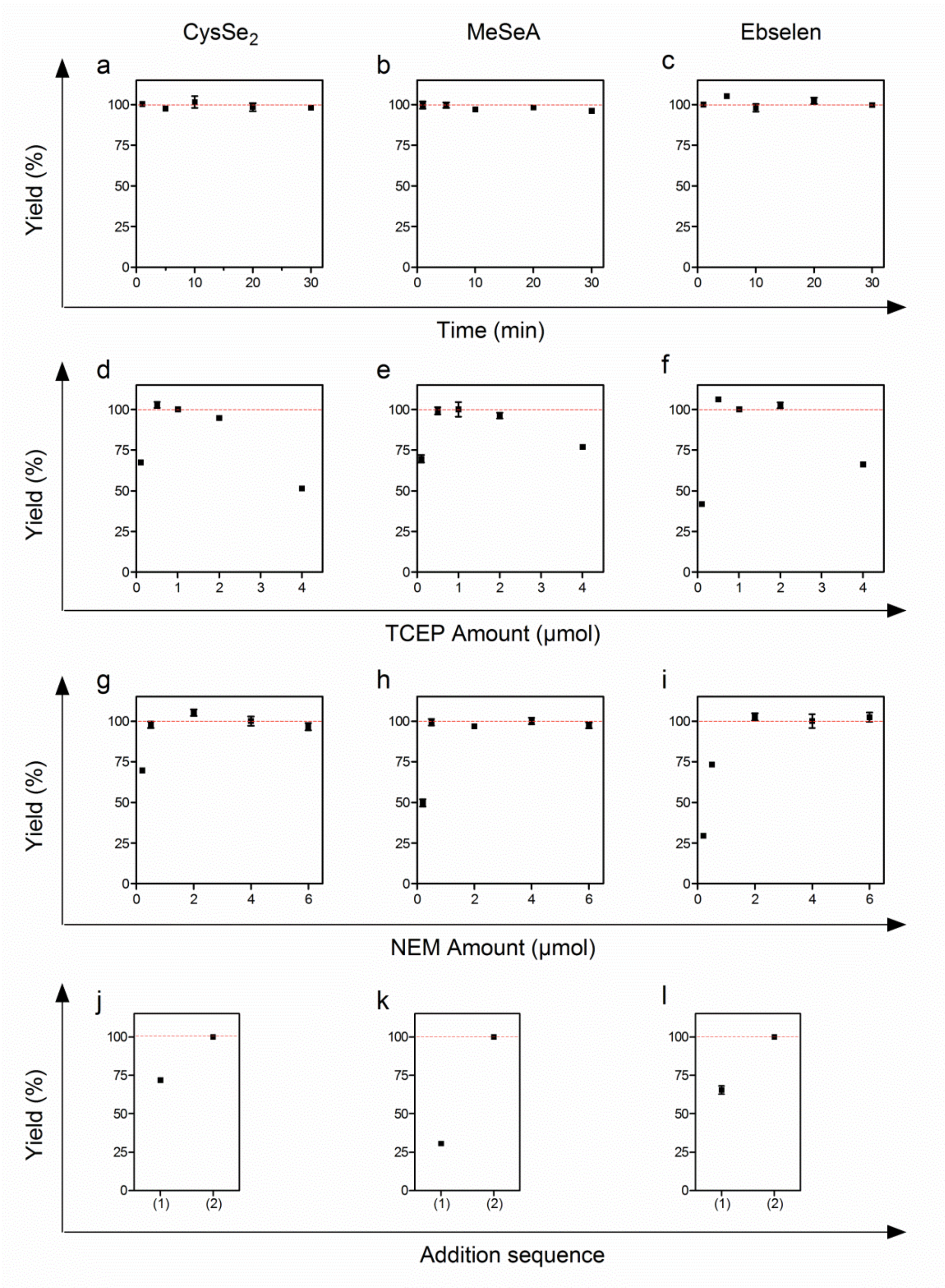

88 **Supplementary Figure 7. LC-MS chromatograms of SeC using RECID.**

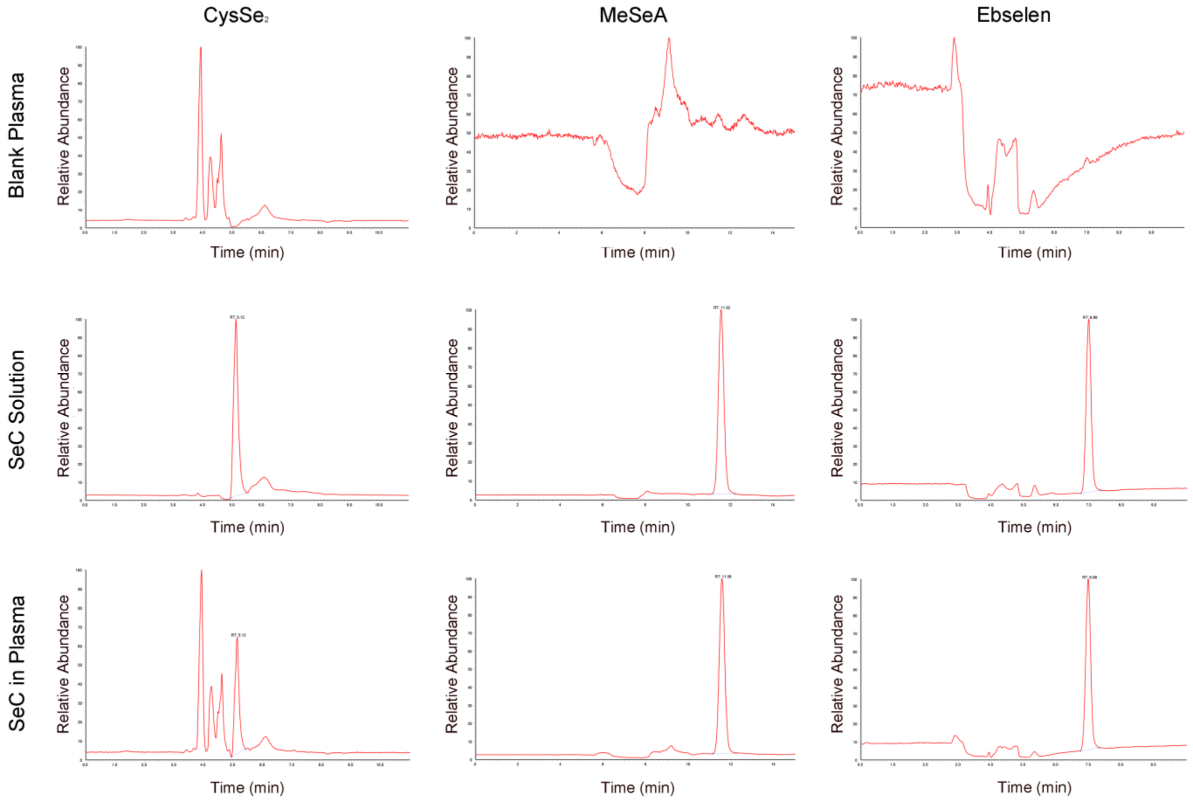


89

**Supplementary Figure 8. Analysis of selenocompound in mouse and human plasma.** SeC was spiked into blank human or mouse plasma (final concentration 10 µg/ml) and analyzed using RECID. Results are shown as the mean ± SD of three technical replicates.

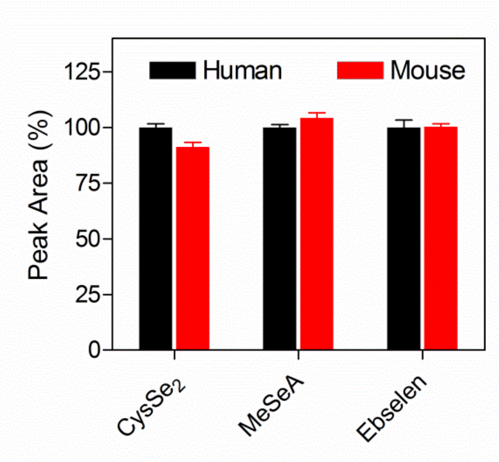

Supplement: Supplementary file 1 — Supplementary information. [file 41598_2020_57983_MOESM1_ESM.pdf]
